# Promoting Return to Work After Vocational Rehabilitation Using a Work-Related Fitness App: Protocol for a Cluster-Randomized Controlled Trial

**DOI:** 10.2196/50200

**Published:** 2024-03-18

**Authors:** David Bühne, Jan Mathis Elling, Christian Hetzel, Torsten Alles

**Affiliations:** 1 Institute for Quality Assurance in Prevention and Rehabilitation (IQPR) German Sport University Cologne Köln Germany

**Keywords:** digital health, eHealth, physical activity, profile comparison, vocational rehabilitation

## Abstract

**Background:**

Retraining programs in vocational rehabilitation are often characterized by a low level of physical activity, even when targeting jobs with primarily physical demands. They might therefore be accompanied by a decline in functional capacity if the lack of physical activity is not compensated by increased activity during leisure time. The implementation of a work-related exercise app might be a promising approach to promoting a return to work in vocational rehabilitation. We developed the “WORKout-app” which provides exercise plans based on a comparison of the physical demands of the retraining profession and the current functional capacity.

**Objective:**

The aim of this study is to examine the effects of app-based exercise during vocational rehabilitation on perceived work ability (primary outcome), occupational self-efficacy, days of sick leave, and return to work (secondary outcomes).

**Methods:**

We conducted a cluster-randomized controlled trial with 2 arms (intervention: WORKout-app vs control: treatment as usual) in 4 cohorts of 5 vocational rehabilitation centers in Germany. Participants are nested within retraining classes per vocational rehabilitation center and per cohort assigned to either the intervention condition or the control condition. The target sample size at the participant level is 598. Measurement time points include baseline, the end of rehabilitation, 3 months after the end of rehabilitation, and 6 months after the end of rehabilitation. Linear and generalized linear mixed-effects models are performed to test for treatment differences in outcomes.

**Results:**

This study is funded by the German Federal Pension Insurance. The trial is registered with the German Clinical Trials Register (DRKS00030775) and approved by the Ethics Committee of the German Sport University Cologne (145/2022).

**Conclusions:**

The findings of the study will inform researchers and practitioners about the effectiveness of an exercise app developed to counteract the effects of physical inactivity during vocational rehabilitation.

**International Registered Report Identifier (IRRID):**

DERR1-10.2196/50200

## Introduction

In 2020, more than 125,000 services for participation in working life were provided by the German Pension Insurance. Of these, 27% among women and 17% among men were educational services. A total of 6 months after finishing, nearly 1 in 2 individuals was either on long-term sick leave (12%), unemployed (22%), receiving further services to promote the participation of disabled people in working life (5%), or receiving a disability pension (4%) [1]. The demands of retraining programs, usually carried out over a period of 12-24 months, are often largely mental in nature, even when targeting jobs with primarily physical work demands. Overall, they are therefore characterized by a low level of physical activity. A lack of physical activity in turn is associated with increased morbidity [2], mortality [3], and low physical capacity [4,5]. Among college students, an association was found between inactivity and self-reported depression, self-harm, and suicidal attempts [6]. A negative perception of the health condition increases the likelihood of failure to return to work [7,8]. These negative effects are not limited to individuals in professions with high physical demands. Even among white-collar workers, higher physical activity is associated with better work ability [9].

Unfortunately, there is no evidence in vocational rehabilitation that a lack of physical activity is commonly compensated by increased activity during leisure time, for example, by using the exercise and sports programs that are offered within the facilities. In contrast, rehabilitants frequently report low levels of physical activity [10]. An increased risk can be assumed for the period of exam preparation in particular. Among college students, physical activity is found to decline significantly in the graduation year [11]. Furthermore, the overall negative impact of the COVID-19 pandemic on physical activity likely amplified this issue [12].

The implementation of work-related functional capacity training, as successfully established in work-related medical rehabilitation, might be a promising approach to promoting a successful return to work. Being a key component of these measures, the demonstrated positive effects of work-related medical rehabilitation on return to work, self-rated work ability [13], and days of sick leave [14] are in large part attributable to the work-related functional capacity training. However, an implementation within vocational rehabilitation would fail as personnel and spatial preconditions are not given. Exercise apps can be considered the opposite in this context. The low personnel and spatial requirements, as well as the possibility of access at any time, constitute their utility and attractiveness. A positive effect on physical activity levels has already been demonstrated meta-analytically for app-based fitness programs [15]. However, there are currently no exercise apps available that are focused on the critical physical demands of work.

The aim of this study is to examine whether providing an app-based exercise intervention focused on the critical physical demands of work can improve the conditions for successful reintegration among individuals in vocational rehabilitation. A cluster-randomized controlled trial is conducted to avoid contamination effects. We hypothesize that participants receiving the job-related exercise app (“WORKout-app”) will report better work ability at the end of rehabilitation as well as 3 and 6 months thereafter compared to participants in the control condition. A further beneficial effect is expected for secondary outcomes, which include occupational self-efficacy, days of sick leave within the follow-up period, and return to work. We hypothesize that these effects are primarily manifested among participants who use the app regularly during the period between the start of the intervention and the end of rehabilitation. Regular use is defined as 12 or more workouts completed in the app.

## Methods

### Study Design

A 9-month cluster-randomized controlled trial with 2 arms (intervention condition vs control condition) is conducted in 5 vocational rehabilitation centers in Germany. In those centers, referral diagnoses are dominated by diseases of the musculoskeletal system and connective tissue, as well as mental and behavioral disorders. The majority of participants are aged between 30 and 49 years and are mostly male candidates [7].

**Figure 1:**
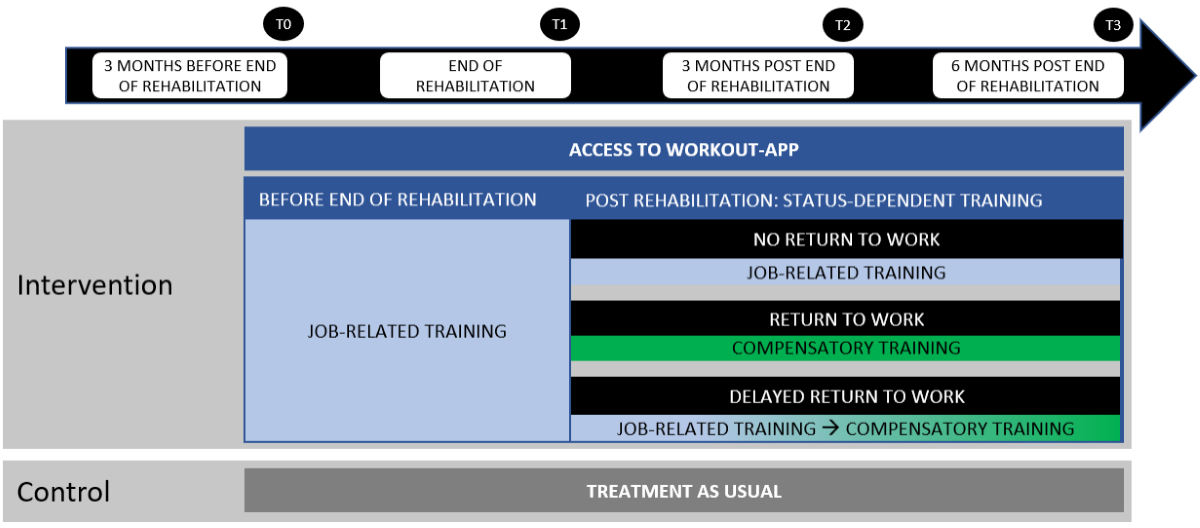
Study design overview.

A flowchart of the study design is presented in Figure 1. Participants in the intervention condition receive an exercise app (“WORKout-app”). Access to the app is provided between 6 and 3 months before the regular end of rehabilitation and ends 6 months after the termination of rehabilitation. Participants in the control condition receive treatment as usual. Participants in both conditions are asked to answer a total of 4 questionnaires. The first questionnaire is distributed at baseline (T0), that is, approximately 3-6 months before the end of rehabilitation. Follow-up questionnaires are distributed at the end of rehabilitation (T1), 3 months post end of rehabilitation (T2), and 6 months post end of rehabilitation. The participating vocational rehabilitation centers (“Berufsförderungswerke”) are located in Berlin/Brandenburg, Cologne, Dortmund, Munich, and Oberhausen.

### Study Sample and Recruitment

We intend to include about 598 participants in total, which provides 80% power (α=.05) to detect a small effect (d=0.25) assuming a design effect of 1.18 (intracluster correlation coefficient=0.02) and a medium cluster size of 10. The sample size was calculated with G*Power (Erdfelder, Faul, and Buchner) [16]. An exclusion criterion at the participant level is a limited ability to exercise safely due to health problems.

### Randomization

Randomization is conducted at the cluster level. Vocational rehabilitation centers in Germany are divided into classes based on the retraining occupations pursued by the rehabilitants. These classes serve as clusters in this study. Whole classes at each vocational rehabilitation center are allocated in a 1:1 ratio to either the intervention or control condition.

Block randomization is carried out within 5 groups of professions that have a similar level of physical work demands. This means that within each group, the classes are randomly assigned to either the intervention or control condition. This procedure is repeated in 4 cohorts per center.

### Measures

#### Primary Outcome

An overview of all constructs and their measurement points is provided in Multimedia Appendix 1. The primary outcome of this study is the perceived work ability, as assessed by the Work Ability Score [17]. This is an 11-point scale used to estimate the current work ability compared to the lifetime best (“Assume that your work ability at its best has a value of 10 points. How many points would you give your current work ability?”).

#### Secondary Outcomes

Secondary outcomes include occupational self-efficacy, days of sick leave within the last 3 months, and return to work (ie, the current employment status). Occupational self-efficacy is assessed using a 6-item scale (eg, “I face difficulties in my job calmly, because I can trust my abilities”), rated on a 5-point response scale ranging from 1 (strongly disagree) to 5 (strongly agree) [18].

#### Characteristics and Covariates

Sociodemographic characteristics include age, gender, migration background, highest school degree, highest vocational qualification, relationship status, and income in the last regularly performed job. Moreover, the county of residence is assessed to determine the regional unemployment rate [19].

The employment biography is assessed by years of employment throughout the entire working life, periods of unemployment as well as periods of sick leave in the 2 years before the start of retraining, and the type of work demand (psychologically demanding, physically demanding, and physically and psychologically demanding) in the last regularly performed job. The retraining profession is divided into 4 categories (commercial and administrative professions, professions in technology and industry, social professions, and electrical and IT professions).

Rehabilitation-related characteristics include the retraining profession, the housing situation (resident of the boarding school vs commuter), and the sponsor of the retraining (German Pension Insurance, Federal Employment Agency, and others). Health-related characteristics include BMI, work ability in relation to physical and mental job demands [17], the type of condition underlying the retraining approval (predominantly physical factors, predominantly psychological factors, physical and psychological factors, and others), general health perception (1=poor to 5=excellent), 5 items of the “Functionality in everyday life” scale of the Indicators of the Rehabilitation Status-24 Questionnaire [20], and the 4-item Patient Health Questionnaire [21]. Furthermore, with regard to the last 3 months, participants are asked whether new health impairments have arisen that have limited their sporting activities.

Physical activity-related characteristics include sports preference (eg, sports disinterested and recreational athletes), sports activity per week, locomotion by bike or on foot in everyday life, as well as the adoption of new exercise or sport habits within the past 3 months. Furthermore, participants’ motives for physical activity are assessed by a selection of items of the Attitude Toward Physical Activity Scales [22,23]. The following motives are assessed: social experience (“…want to be with others”), ascetic experience (“…want to overcome myself”), pursuit of vertigo (“…need excitement and thrill”), health and fitness (“…want to keep myself healthy and fit”), aesthetic experience (“…enjoy aesthetic movements”), and catharsis (“…want to relax”). The items are rated on a 5-point response scale ranging from 1 (strongly disagree) to 5 (strongly agree).

Variables concerning physical activity apps include app experience (eg, “How many physical activity apps have you downloaded so far?”), motives for app use (eg, “I expect a physical activity app to help me reduce or maintain my weight”), the perceived usefulness of physical activity apps (“I consider the use of a physical activity app helpful in achieving my health goals”), the perceived ease-of-use of physical activity apps (“I find it easy to learn how to use a physical activity app”), the social influence regarding the use of physical activity apps (“People whose opinions matter to me would approve of me using a physical activity app”), the behavioral intention to use a physical activity app (“I intend to use a physical activity app in the future”), and technology competence beliefs (“I find dealing with new technology difficult—I just cannot do it most of the time”; eg, [24] and [25]). The items are rated on a 5-point response scale ranging from 1 (strongly disagree) to 5 (strongly agree). The usability of the WORKout-app is assessed by the System Usability Scale [26]. Furthermore, an overall grade for the WORKout-app is measured on a scale ranging from 1 (very bad) to 10 (very good).

Usage behavior is evaluated based on the number of workouts, exercise plans, and self-tests completed. Cut-off values are used to sort out activities in which participants did not actually perform the behavior in question but only clicked through the app (eg, a workout must last at least 10 minutes; otherwise, it does not count). Furthermore, at the completion of each workout, the perceived exertion (“How strenuous did you find this workout?”) is surveyed on a 5-point scale ranging from 1 (too easy) to 5 (too difficult).

With the exception of app usage behavior, all measures are self-reported by respondents.

### Data Analysis

Statistical analyses are performed using R (R Core Team). Descriptive statistics are used to describe the study sample in terms of sociodemographic, vocational, and health characteristics. The primary outcome (ie, work ability) is assessed by linear mixed-effects models. The effect is estimated as the coefficient of the study condition adjusted for the baseline measure, with the follow-up measure as the dependent variable. Random intercepts for the cluster are included. Age, general health perception, retraining profession, and termination of rehabilitation with graduation are included as additional covariates [8]. Participants are nested within retraining classes per vocational rehabilitation center and per cohort assigned to either the intervention condition or the control condition.

Secondary outcomes are assessed in a similar manner, using linear or generalized linear mixed-effects models as appropriate. In the analyses of return to work and days of sick leave, the baseline measures are not included as covariates because there are no baseline measures for these variables. Covariates in the analyses of return to work and days of sick leave include age, general health perception, retraining profession, termination of rehabilitation with graduation [8], regional unemployment rate [19], and periods of unemployment or periods of sick leave in the 2 years before the start of retraining. Covariates in the analysis of occupational self-efficacy include the baseline measure, age, general health perception, retraining profession, termination of rehabilitation with graduation, and the type of condition underlying the retraining approval.

Discontinuation of app use among intervention participants is analyzed by 2 generalized linear mixed-effects models. The first model includes sociodemographic predictors, and the second model additionally includes motives for app use, the system usability scale, and the overall grade.

### Introductory Session and Telephone Support

A joint introductory session (approximately 1-2 hours) led by sports scientists or health psychologists is held as the official start of the intervention in each cluster of the intervention condition. The introductory session begins with an educational part that includes information about the dose-response relationship between physical activity and mortality, the beneficial effects of physical activity on physical and mental health, as well as the recommendations of the World Health Organization regarding physical activity. This educational part aims to raise participants’ awareness and initiate reflection on their own physical activity and the health benefits that can be achieved through increased physical activity. The second and main part of the session is to become familiar with the app and learn how to use its various features in order to enhance participants’ attitudes toward the app and increase their self-efficacy in using it. The session ends with an action planning section, which consists of setting a specific, measurable, attractive, realistic, and time-bound (SMART) goal for training with the app and forming implementation intentions for barriers to training with the app.

During the intervention period, participants are offered weekly opportunities to contact sports scientists within a 2-hour time slot to discuss difficulties and uncertainties in using and training with the app. Participants will be able to get in touch during these time slots by telephone as well as videotelephony on Microsoft Teams (Microsoft Corporation).

### WORKout-App

**Figure 2:**
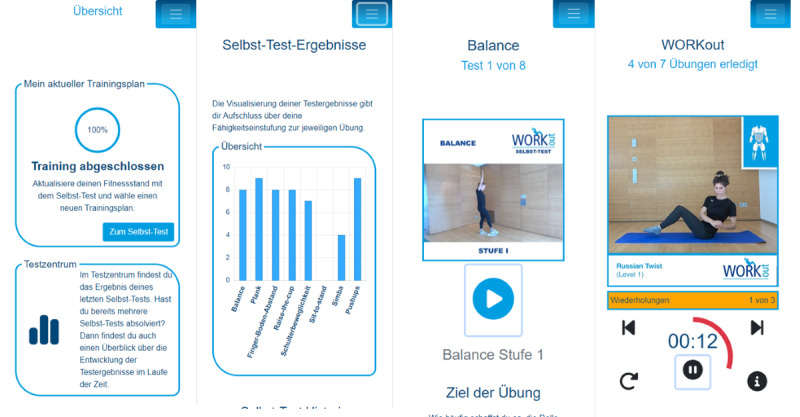
Screens of the WORKout-app used for the intervention.

The WORKout-app is a web app that was created with Blazor (ie, a Microsoft web application framework). Screenshots of the web app are presented in Figure 2. The app contains various exercises that are grouped into workouts, which are in turn integrated into training plans. The primary characteristic of the WORKout-app is that the training is specifically focused on those physical demands of work that could potentially limit return to work in the individual case. The underlying concept is a profile comparison, a comparison of physical abilities and the individual physical demands of the retraining profession. A total of seven characteristics are thereby distinguished: (1) sitting, (2) standing, (3) trunk movements and compulsory trunk postures, (4) demands regarding upper extremities, (5) demands regarding lower extremities including locomotion and endurance, (6) load handling, and (7) balance.

### Classification of Physical Demands of Work

Before the development of the app, the physical demands of work were estimated for all retraining professions according to the 7 characteristics mentioned above. A 6-point scale based on the "Integration von Menschen mit Behinderung in die Arbeitswelt" method [27] was used for this purpose (1=very low demands and 6=demands that require at least above-average abilities).

The individual retraining profession must be specified when setting up the app, which allows for assigning an individual level of physical capacity that should be reached in order to be able to cope with the physical demands of work. Tax clerks, for example, only require a roughly average capacity for sitting, while the other 6 characteristics are weakly pronounced. In the case of in-house technicians, on the other hand, low demands are only given in terms of sitting.

### Physical Capacity Examination

The app contains a self-test with 5 motor tests designed to estimate the ability to cope with the aforementioned physical demands of work. The motor tests include static strength endurance of the trunk (reference for work demands 1 to 3), static strength endurance of the upper extremities (4), physical endurance (5), weight handling (6), and balance (7). The convergent validity of the motor tests was evaluated in advance. The tests for weight handling and balance were strongly correlated with the results of a Functional Capacity Evaluation test [28]. In contrast, only moderate correlations were observed for the remaining motor tests. Given the limited convergent validity, self-ratings are therefore additionally included. A 6-point scale is used for the 5 self-ratings, which are weighted equally with the motor test results.

### Work-Related Training

With regard to all exercises, workouts, and training plans, a distinction is made regarding the expected effect on coping with the physical demands of the work mentioned above. Furthermore, 3 levels of intensity (low, medium, and high) are distinguished. After the completion of the self-test, an automated profile comparison is performed. Depending on the result of this profile comparison, training plans are provided that address those abilities that are below the level required in the profession and that also correspond to the individual's physical capacity. This profile comparison is the key characteristic of the app, characterized by a demand-related orientation toward the individual physical requirements of work. For example, if the profile comparison indicates excessive demands in the characteristic “trunk movements and compulsory trunk postures” and the subject performed roughly average in the self-test, the training plan includes exercises such as back extensions in a lying position and good mornings. If an additional overload were to be expected with regard to the characteristic weight handling, exercises such as curl to press and rowing from a high plank position would also be included. In addition, the training is aimed at those abilities that are poorly manifested (a score of less than 3).

With the transition into work—directly after finishing vocational rehabilitation or in a further course—the workouts turn into compensatory training (Figure 1). The focus shifts to avoiding potential muscle imbalances resulting from the demands of the job. In podiatrists, for example, who are confronted with prolonged sitting as well as stress on the lower back muscles, compensatory training is focused on dynamic exercises of the lower extremities as well as the abdominal muscles.

The information on perceived exertion following completed workouts is used to identify inappropriate training intensities. If participants report twice in a row that a workout was too hard or too easy, they are advised to end the training plan prematurely, after which the training intensity is automatically adjusted.

### Behavior Change Techniques Integrated in the App

The behavior change techniques (BCTs) are described according to the taxonomy of Michie et al [29]. Only the BCTs integrated in the app are described here; the introductory session is described in detail above. The app includes extensive instructions for all exercises in the workouts as well as for all tests of the self-test (BCTs: provide instruction on how to perform the behavior and model or demonstrate the behavior). The instructions are presented in the form of videos in which people perform the exercises or tests (Figure 2), and the execution is explained in detail through voice-over. In the videos of the tests, on-screen text is used sparingly to highlight important issues. In the videos of the exercises, the trained muscle groups are marked in a pictogram of the body. After each completed self-test, the performance in the individual tests is displayed in a graph (Figure 2; BCT: provide feedback on performance). The progress over time can be viewed in separate graphs per test that show the results of the last 5 self-tests. Moreover, a fun feature is implemented in which participants can earn virtual badges for completing workouts and self-tests, as well as for exercising for several weeks in a row (BCT: provide rewards contingent on successful behavior). The app also includes a knowledge section with 12 health- and physical activity-related videos, as well as daily tips and trivia with motivational or scientific content (BCT: provide information on the consequences of behavior in general).

### Ethical Considerations

The trial is registered with the German Clinical Trials Register (DRKS00030775) and approved by the ethics committee of the German Sport University Cologne (145/2022). All participants need to provide written, informed consent to participate.

## Results

This study is funded by the German Federal Pension Insurance. Patient recruitment takes place between March 2023 and July 2024. Considering the follow-up period of 6 months post end of rehabilitation, results are expected to be published in the first quarter of 2026.

## Discussion

The study aims to evaluate the effectiveness of a work-related physical activity intervention through a web app to promote self-reported work ability in vocational rehabilitation. The rationale for the development of the intervention lies in the primarily mental demands of retraining, which favor a decrease in physical performance and thus in the chance of a successful return to work, unless the low demands of retraining are compensated by increased physical activity in leisure time.

User motivation is crucial in digital physical activity interventions [15]. In this study, the timing of receiving the intervention may be a barrier to continued participation. Access to the app is given between 3 and 6 months before the regular end of retraining. This is the period of exam preparation, which has been associated with a decline in physical activity among college students [11]. The focus on the physical demands of work does not necessarily have a positive effect on individual motivation, despite the physiological meaningfulness, if, for example, rehabilitants seek a distance from occupational issues in their leisure time due to a high stress load.

This study underlines the opportunity of adopting holistic approaches in rehabilitation, where physical activity is integrated with innovative digital interventions. Such an approach aligns with the evolving landscape of digitalization. The insights derived from this research are particularly relevant for practitioners and policy makers in the field of vocational rehabilitation. Moreover, the findings may be relevant for digital health interventions in related fields, such as regular vocational training, higher education, or use in aftercare.

## Appendix

Multimedia Appendix 1 Overview of constructs and corresponding measurement points.

## Abbreviations

BCT: behavior change technique
SMART: specific, measurable, attractive, realistic, and time-bound

## Data Availability

Data sharing is not applicable to this research protocol as no data sets were generated or analyzed.
