# Supplementary material for: Promoting Return to Work After Vocational Rehabilitation Using a Work-Related Fitness App: Protocol for a Cluster-Randomized Controlled Trial
Source: JMIR Res Protoc. 2024 Mar 18;13:e50200. doi: 10.2196/50200 (PMC10985606; doi:10.2196/50200)
Supplement: Multimedia Appendix 1 [file resprot_v13i1e50200_app1.docx]

# Appendix A – Overview of Constructs and Corresponding Measurement Points

| Sociodemographic characteristics / Employment biography | T^0^ | T^1^ | T^2^ | T^3^ |
| --- | --- | --- | --- | --- |
| - Age - Gender - Migration background - Highest school degree - Highest vocational qualification - Relationship status - County of residence | X |  |  |  |
| - Years of employment throughout the entire working live - Periods of unemployment in the two years prior to the start of vocational rehabilitation - Periods of sick leave in the two years prior to the start of vocational rehabilitation - Type of work demand in the last regularly performed job | X |  |  |  |
| - Income in the last regularly performed job | X |  |  | X |
| - Return to work (current employment status) - Days of sick leave |  | X | X | X |
| - Termination of rehabilitation with graduation |  | X | X |  |

| Rehabilitation-related characteristics / Health-related characteristics / Physical activity-related characteristics | T^0^ | T^1^ | T^2^ | T^3^ |
| --- | --- | --- | --- | --- |
| - Work Ability Score - Work ability in relation to physical and mental job demands - Occupational self-efficacy | X | X | X | X |
| - Occurrence of new health impairments within the last three months with limiting effect regarding sports activities |  | X | X | X |
| - Retraining profession - Housing situation - Sponsor of the retraining | X |  |  |  |
| - Body mass index - Type of condition underlying the retraining approval - General health perception - 4-item Patient Health Questionnaire | X |  |  |  |
| - Five items of the "Functionality in everyday life"-scale of the IRES-24 Questionnaire - Sports activity per week | X | X | X | X |
| - Sports preference - Locomotion by bike or on foot in everyday life - Motives for physical activity | X |  |  |  |
| - Adoption of new exercise or sport habits within the past three months |  | X | X | X |

| Fitness app-related characteristics / Usage behavior | T^0^ | T^1^ | T^2^ | T^3^ |
| --- | --- | --- | --- | --- |
| - App experience - Motives for app use - Perceived usefulness of physical activity apps - Perceived ease-of-use of physical activity apps - Social influence regarding the use of physical activity apps - Behavioral intention to use a physical activity app - Technology competence beliefs | X |  |  |  |
| - Overall grade for the WORKout-app - System Usability Scale |  | X |  |  |
| - Number of workouts, exercise plans, and self-tests completed | X | X | X | X |
